# Sensitization of Cancer Cells to Radiation and Topoisomerase I Inhibitor Camptothecin Using Inhibitors of PARP and Other Signaling Molecules

**DOI:** 10.3390/cancers10100364

**Published:** 2018-09-28

**Authors:** Yusuke Matsuno, Mai Hyodo, Haruka Fujimori, Atsuhiro Shimizu, Ken-ichi Yoshioka

**Affiliations:** 1 Division of Carcinogenesis and Cancer Prevention, National Cancer Center Research Institute, 5-1-1 Tsukiji, Chuo-ku, Tokyo 104-0045, Japan; yumatsun@ncc.go.jp (Y.M.); mhyodo@ncc.go.jp (M.H.); harfujim@ncc.go.jp (H.F.); davanauma@gmail.com (A.S.); 2 Department of Applied Chemistry, Faculty of Science, Tokyo University of Science, 1-3 Kagurazaka, Shinjuku-ku, Tokyo 162-8601, Japan; 3 Biological Science and Technology, Tokyo University of Science, 6-1-1 Niijuku, Katsushika-ku, Tokyo 125-8585, Japan

**Keywords:** chemotherapy, anticancer drug, camptothecin, PARP inhibitor, radiotherapy, sensitizer, DNA repair, apoptosis, checkpoint response

## Abstract

Radiation and certain anticancer drugs damage DNA, resulting in apoptosis induction in cancer cells. Currently, the major limitations on the efficacy of such therapies are development of resistance and adverse side effects. Sensitization is an important strategy for increasing therapeutic efficacy while minimizing adverse effects. In this manuscript, we review possible sensitization strategies for radiation and anticancer drugs that cause DNA damage, focusing especially on modulation of damage repair pathways and the associated reactions.

## 1. Introduction

Radiation and anticancer drugs that damage DNA were developed many years ago, and are still widely used for cancer therapy [1,2]. These methods achieve their clinical efficacy by promoting the induction of apoptosis in response to DNA damage and cellular stress [3,4,5,6]. A major problem that arises when using such anticancer drugs is development of resistance, which causes treatment to fail [7,8,9,10]. Other major problems include side effects, in which toxicity in a non-targeted tissue limits the tolerable dosage, thereby decreasing the therapeutic efficacy and leading, ultimately, to recurrence [11,12]. The existence of these problems emphasizes the importance of using sensitizers to efficiently induce cancer cell death [11]. Sensitization strategies include combination therapies with multiple drugs, which can achieve synergistic induction of apoptosis in cancer cells.

One important strategy for sensitizing cancer cells to radiation or DNA-damaging drugs is modulation of DNA repair pathways. For example, susceptibility to the DNA-damaging agent cisplatin is higher in cells harboring mutations in *BRCA1*, *BRCA2*, and *Rad51*, which cause deficiencies in homologous recombination (HR) [13,14]. In fact, even in such HR-defective backgrounds, damaged cells exhibit normal checkpoint responses [14], allowing them to induce apoptosis when they sense DNA damage. Such an effect could be produced by simultaneous administration of multiple drugs, as when a poly (ADP-ribose) polymerase (PARP) inhibitor is used to sensitize cells to the DNA methylation agent temozolomide [15,16,17].

In this manuscript, we review recently developed methods for efficiently inducing cancer cell death, focusing on sensitization strategies for chemotherapy with camptothecin (CPT) and radiotherapy. Many of these strategies are based on the modulation of DNA repair pathways. In addition, we draw special attention to mechanistic insights.

## 2. PARP Inhibitor as a Potential Sensitizer to Top1 Inhibitor

### 2.1. Top1 Inhibitor Treatment in the Presence of PARP Inhibitor

**Figure 1:**
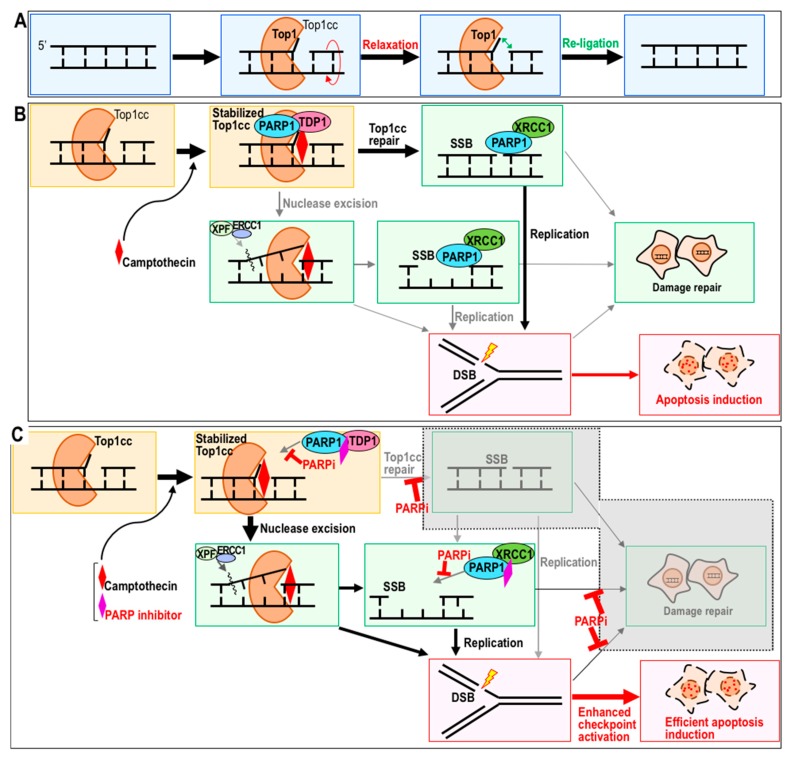
Model of the topoisomerase 1 reaction and its inhibition by camptothecin (CPT) and poly (ADP-ribose) polymerase (PARP) inhibitor. (**A**,**B**) Top1 cuts a single strand of DNA to relax super-coiled DNA stress (**A**). CPT blocks the ligation step and, hence, induces toxicity during the subsequent S phase in association with replication stress (**B**). (**C**) PARP inhibitor sensitizes the cell to CPT by blocking multiple steps of the repair pathway. In this cellular background, apoptosis is induced more efficiently.

Topoisomerase 1 (Top 1) is an enzyme that cuts one strand of the DNA duplex and religates the broken ends to relax DNA supercoiling stress, which often arises when DNA or RNA polymerases are operating [18,19] (Figure 1A). CPT is a naturally occurring Top1 inhibitor isolated from *Camptotheca acuminate* [20]. Derivatives of CPT, such as topotecan and irinotecan, are widely used for cancer chemotherapy [21]. Multiple studies show that PARP inhibitors are potential sensitizers for chemotherapy with CPT, based on the fundamental observation that induction of apoptosis by CPT in vitro is stronger when cells are simultaneously treated with PARP inhibitor [22,23]. To efficiently induce the desired therapeutic outcomes while minimizing side effects, it is important to carefully determine how sensitization is achieved. Recent work showed that, when a PARP inhibitor is administered simultaneously with CPT or its derivatives, multiple reaction steps are modulated [24,25] (Figure 1B,C).

CPT (or its derivatives) binds to the Top1–DNA cleavage complex (Top1cc) and inhibits the religation step (Figure 1B). Top1cc is trapped and stabilized by CPT, as well as by endogenous DNA lesions, including a basic sites, mismatches, oxidized bases, and nicks [26,27]. Therefore, Top1cc, like the intermediates of its repair process, is a cause of DNA double-strand break (DSB) formation, mainly during replication stress arising during the subsequent S phase (Figure 1B). Removal of Top1cc can be mediated by either PARP–TDP1 (tyrosyl-DNA phosphodiesterase 1) complexes or the XPF (xeroderma pigmentosum complementation group F)-ERCC1 (Excision repair cross-complementing group 1) endonuclease [28,29] (Figure 1B); consequently, when CPT is administered in the presence of PARP inhibitor, one of the major pathways is blocked.

In the presence of PARP inhibitor olaparib, Top1cc stably accumulates after CPT treatment [30] (Figure 1C). Moreover, the rate of DSB formation caused by CPT treatment is dramatically elevated in the presence of PARP inhibitor. Although replication stress-associated DSBs caused by CPT are primarily targeted by HR, induction of HR is suppressed in the presence of PARP inhibitor [25]. In addition, microhomology-mediated end joining (MMEJ) is also blocked by PARP inhibitor [31]. Therefore, non-homologous end joining (NHEJ) is the only repair pathway available to the cell under these conditions. The checkpoint response is much more effectively activated in cells treated with CPT and PARP inhibitor together, than in cells treated with CPT alone; consequently, dual treatment leads to more effective induction of apoptosis [32,33]. Thus, PARP inhibition causes multiple effects, probably because PARP1 and 2 mediate multiple repair pathways, including base excision repair, MMEJ, and HR [34,35,36].

### 2.2. Sensitization to CPT by PARP Inhibitor

**Figure 2:**
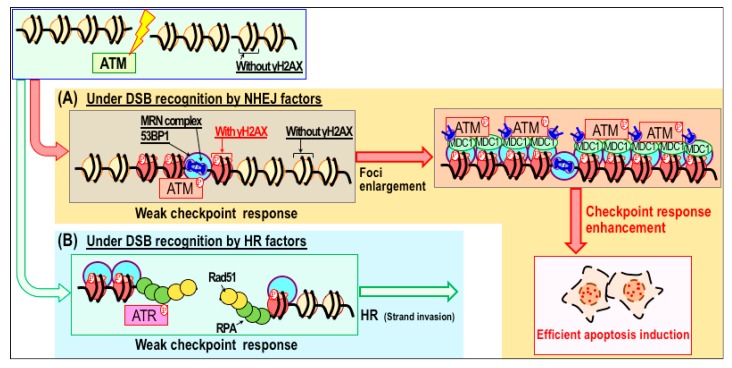
Model of checkpoint response enhancement through modulation of DNA repair pathways. (**A**,**B**) In response to double-strand breaks (DSBs), γH2AX/53BP1 foci form immediately, and are subsequently enlarged in association when the damage checkpoint response is stimulated (**A**). Under these conditions, repair factors associated with non-homologous end joining (NHEJ) accumulate at DSB sites. Stimulation of the checkpoint response increases the efficiency of apoptosis induction. By contrast, DSBs recognized by homologous recombination (HR) factors are usually not associated with the enlargement of γH2AX foci or stimulation of the damage checkpoint response (**B**).

Given that PARP inhibitors cause multiple effects in CPT-treated cells, it is important to determine which of these effects is critical for sensitization. Recent studies showed that the PARP inhibitor ABT-888 (veliparib) increases CPT-induced cytotoxicity by mediating DSB accumulation, without increasing the level of Top1cc [29]. This implies that CPT sensitization by PARP inhibitor is correlated with DSB accumulation, but not directly associated with stabilization or accumulation of Top1cc. In mechanistic terms, sensitization could be mediated by promotion of the associated checkpoint response, leading to more effective induction of apoptosis [37]. DSBs caused by CPT in the presence of PARP inhibitor are targeted by NHEJ factors. In particular, enlargement of γH2AX/p-ATM foci are often observed in association with heightened damage checkpoint signaling, resulting in more efficient induction of apoptosis (Figure 2). By contrast, these features are not effectively activated during HR, which is usually triggered when CPT is administered alone. Given that apoptosis is induced as a consequence of damage checkpoint signaling [38,39,40], it is reasonable to expect that apoptosis would be strongly induced when checkpoint signaling is strongly activated.

In support of this hypothesis, synthetic lethality is induced by pharmacologic inhibition of PARP1/2 in HR-defective cancer cells. It is well established that the PARP inhibitor olaparib (or veliparib) selectively kills BRCA1/2-mutated breast and ovarian cancers [22,23,41,42]. In the presence of PARP inhibitor, these cells spontaneously accumulate DSBs. Since those DSBs are not efficiently repaired by HR or MMEJ (due to the presence of the BRCA1/2 mutation and inhibition of PARP), NHEJ is the only pathway available to repair those DSBs [43]. However, despite having functional NHEJ, cells in this context undergo apoptosis rather than repair, analogous to the situation in cells treated with CPT in the presence of PARP inhibitor (Figure 2).

### 2.3. Potential Combination Therapy with CPT and a PARP Inhibitor as a Sensitizer

Although combination treatment with CPT (or its derivatives) and olaparib effectively induces cancer cell killing in vitro, a phase I study concluded that this combination is not suitable for clinical use, due to dose-limiting adverse effects, causing the maximum tolerated dose to be subtherapeutic [44]. The main dose-limiting adverse effects were neutropenia and thrombocytopenia, as previously reported for topotecan treatment [45], but these toxicities were observed at substantially lower doses of both drugs [44]. Thus, combination therapy with CPT (or its derivatives) and olaparib has, thus far, failed as a strategy for cancer chemotherapy. However, a series of studies using this combination demonstrated that modulation of repair pathways is conceptually useful as a strategy for efficient induction of apoptosis when cells are treated with DNA-damaging agents.

## 3. Radiation Therapy and Its Sensitizers

### 3.1. Radiosensitizers and Their Clinical Use

Clinical trials of radiosensitizers for various cancers have been reported; these trials were based on improvements in killing efficiency in vitro [46,47,48,49]. Examples of increased killing efficiency include radiosensitization of glioblastoma using temozolomide (DNA alkylating agent) [50], prostate cancer using gefitinib (EGFR inhibitor) [51], and non-small-cell lung cancer (NSCLC) using paclitaxel (mitotic inhibitor) [46,52,53]. However, combination therapy has some limitations; for example, elderly NSCLC patients cannot tolerate standard chemoradiation regimens [46]. Survival rates after combined use of radiotherapy plus gefitinib are better that those after radiation therapy only; the most common dose-limiting toxicity is a grade 3 to 4 increase in transaminase activity [51]. Nevertheless, it is important to gain mechanistic insight to improve the therapeutic efficiency of combined therapy in general.

### 3.2. Sensitization to Radiation Therapy through Modulation of Repair Pathways

Radiation exposure primarily causes DNA damage. Therefore, as with anticancer drugs that cause DNA damage, its therapeutic effects are mainly due to induction of apoptosis in response to DSBs. One strategy for sensitizing cancer cells to radiation therapy is modulation of DNA repair pathways. For example, the chemotherapeutic drug cisplatin, which crosslinks DNA strands, is used in conjunction with ionizing radiation (IR) to treat various types of cancer, including cervical carcinomas, and head and neck cancers [54,55]. Cells can be sensitized to cisplatin through inhibition of NHEJ [56], conceptually analogous to the aforementioned sensitization to DNA-damaging anticancer drugs through the modulation of repair pathways [32,33,43].

DNA-dependent protein kinase (DNA-PK) and PARP inhibitors also increase the cytotoxicity of radiation [17,57,58,59,60]. In the presence of PARP inhibitor, damage checkpoint activation in response to radiation exposure is significantly elevated [61], leading to efficient induction of cancer cell death through apoptosis. Radiosensitization by PARP inhibition is primarily due to suppression of PARP-mediated repair pathways, analogous to PARP inhibitor-mediated sensitization to CPT [32,33]. Thus, repair pathway modulation is a feasible strategy for sensitization to radiotherapy. This idea is further supported by the observation that inhibition of DNA-PK, which inhibits NHEJ, also increases the cytotoxicity of radiation [57,58].

IR causes multiple types of DNA damage, including DSBs and single-strand breaks (SSBs). IR also causes formation of reactive oxygen species (ROS) which, in turn, promote production of oxidized nucleotide adducts, such as 8-oxoguanine [62,63,64]. In addition, SSBs and ROS cause replication stress, which is itself associated with formation of DSBs [65]. Although apoptosis can be induced in response to DSBs [66], the DSBs caused directly by therapeutic radiation are usually repaired within a few hours [67]. In mechanistic terms, it remains unclear which damage, stresses, and adducts make the greatest contributions to cancer cell killing. A recent study revealed that persistent DSBs in irradiated cells form in association with replication stress during the S phase following the repair of radiation-induced DSBs [68], suggesting that these later DSBs are primarily responsible for cytotoxicity. In this case, the sensitization effect caused by PARP inhibitor might be identical to that observed during CPT sensitization.

### 3.3. Radiotherapy in Conjunction with Molecularly Targeted Agents and Immune Checkpoint Inhibitors

Some molecularly targeted agents efficiently sensitize specific types of cancer to radiation. For example, cetuximab, an anti-epidermal growth factor receptor (EGFR) antibody, is currently used as a sensitizer for radiation therapy in cases of head and neck squamous cell carcinoma. The sensitization effect is induced by inhibiting the radiation-induced upregulation of HIF-1α [69], suggesting that modulation of growth factor signaling represents another potential target for sensitization to radiation therapy.

Radiotherapy might also be complemented by immune checkpoint blockade [70,71]. Radiation, initially thought to be an immunosuppressive, was recently shown to be a promising candidate for such a combination [72]. Victor et al. demonstrated that radiation therapy, in conjunction with anti-cytotoxic-T lymphocyte-associated protein 4 (CTLA4) antibody, is more effective than either treatment alone [72]. Furthermore, addition of anti-prognostic of programmed cell death ligand 1 (PD-L1) further suppresses the adaptive immune resistance that arises in patients treated with radiation therapy and anti-CTLA4 antibody [73]. These observations indicate that the combination of radiation therapy with dual immune checkpoint blockade represents a promising strategy for radiotherapy sensitization. In the future, it may be possible to further develop sensitization strategies by combining multiple sensitizers.

### 3.4. Radiosensitivity by Autophagy Regulatory Drugs

Multiple studies show that radiation sensitivity is associated with the activation status of autophagy, which is normally involved in maintaining intracellular metabolic balance [74,75]. Although it remains unclear how autophagy is associated with radiation sensitivity, it is clear that autophagy is induced by radiation. Moreover, it is further activated by simultaneous treatment with DNA-PK and PARP inhibitors, cisplatin, and anti-EGFR antibody [60,75,76,77]. Importantly, autophagy activation is associated with the efficiency of cancer cell killing [78]. However, in some other contexts, radioresistance is promoted when autophagy is activated [79]. Thus, although autophagy activation is usually associated with radiosensitivity, it can also induce resistance. Currently, we do not have a way to simultaneously control the cancer-suppressing and -promoting effects of autophagy [80].

### 3.5. Proton Beam and Carbon-Ion Beam Therapy

Proton beam therapy (PBT) and carbon-ion beam radiotherapy are more recent modes of radiation therapy; both have fewer adverse effects and higher therapeutic efficacy than conventional radiation therapy [81]. Like radiation therapy, PBT also causes DNA damage, leading to induction of apoptosis in tumor cells [82]. The advantage of PBT is that the proton beam penetrates deeply into tissues, and can be controlled to efficiently target tumors while reducing exposure of the surrounding normal tissues. Carbon-ion beam therapy also has several advantages over radiotherapy; these include higher relative biological effectiveness, lack of an oxygen effect, and lower cell cycle-related radiosensitivity [83,84]. Importantly, carbon-ion beams have a marked killing effect even on cancer cells that are resistant to X- and γ-radiation [85]. The increased killing effect appears to be due to complex DSBs caused by the high linear energy transfer effect of carbon ions [86]. Such complex DSBs are much harder to repair than DSBs caused by X- or γ-radiation [87,88]. Such differences in repair efficiency might be due to differences in the repair mechanisms involved. Damage caused by proton- and carbon-ion radiation is repaired mainly by NHEJ; however, damage caused by the latter is also repaired by HR [89].

Sensitization studies were also reported for hadron radiation. Killing effect of cancer cells by carbon-ion beam irradiation was enhanced by carboplatin (cisplatin analogue) [90], paclitaxel [90], PU-H71 (HSP90 inhibitor) [91], and genistein (isoflavone compound) [92]. Vorinostat (histone deacetylase inhibitor) can sensitize cancer cells to proton beam and carbon ion beam radiations as well as γ-radiation [93]. Currently, several clinical trials involving combinations of these two radiation modalities plus chemotherapeutic agents are ongoing.

## 4. Conclusions and Prospects

Radiotherapy and chemotherapy with drugs that damage DNA are commonly used to treat cancer. However, the therapeutic efficacy of such approaches is often limited by multiple adverse effects. Recent studies suggest various strategies for sensitization, including modulation of DNA damage repair pathways and immune checkpoint blockade. Since it is difficult to predict side effects, it is important to carefully investigate the mechanisms underlying each sensitization strategy, both individually and in combination.
